# Sex differences and gender-related disparities in transcatheter tricuspid edge-to-edge repair for secondary tricuspid regurgitation: toward a personalized approach in cardiac care

**DOI:** 10.3389/fcvm.2026.1831980

**Published:** 2026-10-30

**Authors:** Gianluca Pagnoni, Francesco Sbarra, Arianna Maini, Ashraf Nassar, Simona Castellino, Francesco Marangi, Damiano De Cesare, Martina Moretti, Pierluca Macripò, Marco Loffi, Giulia Ghizzoni, Dalia Caleffi, Federica Arturi, Gabriele Melegari, Susan Darroudi, Alessio Baccarani, Fabio Alfredo Sgura, Anna Vittoria Mattioli, Daniela Aschieri, Francesco Fedele, Carlo Mario Lombardi, Marianna Adamo, Alberto Barbieri, Francesca Coppi

**Affiliations:** 1 Cardiologic Unit, Specialistic Medicine Department, Guglielmo da Saliceto Hospital, Piacenza, Italy; 2 National Institute for Cardiovascular Research (INRC), Bologna, Italy; 3 Cardiology Division, Department of Biomedical Metabolic and Neural Sciences, University of Modena and Reggio Emilia, Modena, Italy; 4 Division of Cardiology, Cremona Hospital, Division of Cardiology, Azienda Socio-Sanitaria Territoriale di Cremona, Cremona, Italy; 5 Cardiology Division, Azienda Ospedaliera Universitaria di Modena, Modena, Italy; 6 School of Anaesthesia and Intensive Care, University of Modena and Reggio Emilia, Modena, Italy; 7 Department of Medical and Surgical Sciences for Children and Adults, University of Modena and Reggio Emilia, Modena, Italy; 8 Department of Quality of Life, University of Bologna - Alma Mater Studiorum, Bologna, Italy; 9 Emeritus Professor of Cardiology, Sapienza University of Rome, Rome, Italy; 10 Department of Medical and Surgical Specialties, Radiological Sciences, and Public Health, Institute of Cardiology, ASST Spedali Civili, University of Brescia, Brescia, Italy

**Keywords:** cardiac care, sex, transcatheter tricuspid valve, tricuspid regurgitation, ventricular dilation

## Abstract

**Background:**

Tricuspid regurgitation (TR) is a frequently underdiagnosed and undertreated condition that is linked to a poor prognosis. Tricuspid regurgitation (TR) may occur as atrial secondary TR (A-STR), which is associated with right atrial dilation and is more common in women, or as ventricular secondary TR (V-STR), which is associated with right ventricular dilation and is more frequently observed in men. Transcatheter edge-to-edge repair (TEER) of tricuspid regurgitation using the TriClip system (Abbott Cardiovascular) has demonstrated a favorable procedural safety profile and consistent improvements in TR severity, symptoms, and quality of life in selected patients at high or prohibitive surgical risk. Randomized 1-year trials did not show significant reductions in mortality or heart failure hospitalization; however, the subsequent 2-year TRILUMINATE follow-up reported fewer recurrent heart failure hospitalizations without a mortality benefit.

**Objective:**

To review evidence on sex differences in tricuspid regurgitation across epidemiology, pathophysiology, guideline-directed management, and outcomes after T-TEER, together with gender-related disparities in access to care, in order to inform more personalized therapeutic strategies.

**Methods:**

A structured narrative review of PubMed/MEDLINE was conducted from database inception through 31 January 2026. The electronic search was supplemented by backward citation searching and by the examination of relevant guidelines and consensus documents from official scientific society websites. Study identification and selection were documented using a PRISMA-informed flow diagram. Because of substantial clinical and methodological heterogeneity among the available studies, the evidence was synthesized narratively without quantitative meta-analysis.

**Evidence:**

Randomized trials and registries demonstrate high rates of successful device implantation and tricuspid regurgitation reduction after tricuspid transcatheter edge-to-edge repair, although procedural endpoints, definitions, denominators, and assessment time points differ across studies and are therefore not directly comparable. At 1 year, the mean change in Kansas City Cardiomyopathy Questionnaire score from baseline was +12.3 points with T-TEER versus +0.6 points with medical therapy in the TRILUMINATE primary randomized cohort, and +15.9 versus +0.4 points in Tri.Fr, corresponding to reported between-group differences of +11.7 and +14.5 points, respectively. In the single-arm bRIGHT registry, the paired within-group mean KCCQ improvement from baseline was +19 points. Exploratory observational data suggest that sex may modify the prognostic association between right ventricular–pulmonary arterial coupling and mortality after TTVI; however, the proposed sex-specific TAPSE/mPAP thresholds were internally derived in a single retrospective cohort and require external validation before any clinical application.

**Conclusion:**

TEER with the TriClip system appears to be a viable treatment option in women, who often present with A-STR and preserved right ventricular function. Available evidence suggests broadly comparable procedural success and symptomatic improvement in women and men, although most sex-specific findings derive from exploratory analyses. Integrating sex-specific considerations into diagnosis, treatment, and follow-up may help optimize intervention timing and improve the overall management of TR.

## 1 Introduction

### 1.1 Tricuspid regurgitation: underestimated clinical burden and growing therapeutic relevance

Tricuspid regurgitation (TR) has long been overlooked as a cardiovascular condition, historically considered secondary to other valvular diseases with limited therapeutic options. However, recent scientific evidence has changed this perception, revealing that severe tricuspid regurgitation carries significant prognostic implications. Its prevalence is on the rise, especially among the aging population, necessitating increased attention in both diagnosis and therapeutic settings (1, 2).

Recent studies have shown that severe TR is associated with an increased risk of morbidity and mortality, with a direct impact on quality of life and long-term outcomes (3).

In particular, TR contributes to the progression of right heart failure, systemic congestion, and reduced exercise tolerance, representing an independent prognostic factor for adverse outcomes.

Despite growing recognition of its prognostic impact, TR remains undertreated, even in specialized centers, largely due to concerns regarding procedural risk and the historical limitations of surgical treatment strategies (2, 3). However, advancement in pathophysiological knowledge and the emergence of transcatheter techniques are changing this scenario, opening new therapeutic possibilities for a condition that was previously considered “orphan” of effective treatments.

### 1.2 Triclip: evolution of treatment options and impact on vulnerable populations

The therapeutic landscape for severe tricuspid regurgitation has evolved considerably, underscoring the importance of early diagnosis and timely intervention. Alongside advances in surgical techniques, innovative percutaneous approaches have expanded treatment options for patients previously considered inoperable or at prohibitive surgical risk (4). Transcatheter tricuspid valve intervention (TTVI) encompasses a spectrum of percutaneous strategies, including edge-to-edge repair systems (such as TriClip and other coaptation devices) and emerging transcatheter tricuspid valve replacement (TTVR) platforms. While edge-to-edge repair currently represents the most established approach—largely driven by the TriClip experience—the broader field of TTVI continues to evolve, with multiple device concepts under active clinical investigation.

Among these innovations, transcatheter edge-to-edge repair—most extensively studied with the TriClip system (Abbott Cardiovascular)—has emerged as a key therapeutic option. This minimally invasive approach has been associated with significant reduction in tricuspid regurgitation severity and improvement in heart failure symptoms, including in high-risk populations such as elderly patients with multiple comorbidities (4). Data from the TR-Interventional Study and the Italian TRIS registry further support improvements in functional class, quality of life, and reductions in hospitalization rates (5). These findings are further supported by prospective studies and international registry data. TRILUMINATE and the initial 106-patient TriValve registry demonstrated the feasibility of transcatheter tricuspid repair and early symptomatic improvement, although the initial TriValve report provided only short-term, non-sex-stratified outcomes (6, 7).

The randomized TRISCEND II trial, including 400 patients, showed that TTVR with the EVOQUE system plus medical therapy was superior to medical therapy alone for the hierarchical primary endpoint, although the benefit was driven primarily by improvements in symptoms and quality of life. Women represented approximately three quarters of the study population, but sex-specific outcome, device-sizing, and anatomical screening analyses were not reported (8). However, sex-stratified outcome data for these devices remain insufficient for a meaningful comparison between women and men. Accordingly, although TTVR is acknowledged as an important and rapidly evolving component of the transcatheter tricuspid field, the present review specifically focuses on transcatheter tricuspid edge-to-edge repair (T-TEER), for which the largest body of sex-specific clinical evidence is currently available.

Early intervention with edge-to-edge repair may offer advantages over conservative treatment. This is particularly relevant in fragile patients, who represent an increasing proportion of individuals affected by tricuspid regurgitation. This approach enables earlier intervention in the disease course, potentially before the onset of advanced right heart failure, and has been associated with meaningful improvements in symptoms and quality of life (1, 9).

Available evidence indicates that T-TEER has a favorable procedural safety profile in carefully selected high-risk patients. However, randomized trials have compared T-TEER with medical therapy rather than with surgery; therefore, these data do not establish a direct reduction in peri-procedural risk compared with surgical treatment (9, 10). However, despite these advances, several challenges persist, particularly regarding patient selection and the refinement of therapeutic strategies to improve clinical practice, requiring further implementation of available scientific data and updates of current guidelines.

As transcatheter therapies expand, having a deeper understanding whether disease phenotype, referral patterns, and treatment response differ between women and men becomes increasingly relevant.

### 1.3 Focus on gender medicine: motivations, emerging evidence, and objectives of this review

Sex- and gender-informed medicine has gained importance in valvular heart disease, highlighting both sex-related biological differences and gender-related disparities that may influence the clinical presentation, diagnosis, and management of tricuspid regurgitation (TR).

In this review, *sex* refers to biological attributes (e.g., anatomy, physiology, hemodynamics, and biomarker patterns) that may influence TR phenotype, echocardiographic parameters, and response to intervention. *Gender* refers to sociocultural and structural factors (e.g., referral pathways, access to specialist evaluation, symptom reporting, and workforce representation) that may contribute to disparities in diagnosis and treatment. Where available, we report evidence stratified by sex; we use gender when discussing access- and system-related inequities.

Recent evidence shows that women often present at a more advanced stage of the disease, with higher comorbidity, particularly pulmonary hypertension, and increased susceptibility to adverse events, including worsening of right heart function and reduced survival.

An important aspect concerns the different etiological distribution. Women appear more likely to present with atrial secondary tricuspid regurgitation (A-STR), a phenotype characterized by tricuspid annular dilatation driven predominantly by right atrial remodeling and frequently associated with atrial fibrillation. However, the magnitude of this sex difference varies according to cohort selection and phenotype definition. In a multicenter cohort of 702 patients undergoing transcatheter tricuspid valve intervention, A-STR was identified in 41.7% of women and 24.4% of men. Thus, the available evidence supports a substantial female predominance but not a female-exclusive phenotype (1, 11, 12).

Limited post-mortem evidence suggests possible sex-associated differences in atrioventricular annular size and tissue composition. However, these observations derive from a single anatomical series, and their generalizability and *in vivo* relevance to tricuspid annular remodeling and A-STR remain uncertain. They should therefore be regarded as hypothesis-generating rather than as established mechanisms explaining sex-related differences in TR progression (13–15).

Women with tricuspid regurgitation are typically diagnosed and treated at later stages compared to men. They often present with a higher NYHA class, greater systemic congestion, and poorer functional status. This delay in diagnosis and treatment may indicate a more gradual clinical course of female TR. It is often associated with heart failure with preserved ejection fraction (HFpEF) and atrial fibrillation, conditions that can obscure the progression of disease (16–19).

In the TRILUMINATE trial, women accounted for 54% of the enrolled population, representing an important step toward better sex representation in TTVI studies. Subgroup analyses suggested differences in clinical response between sexes, with a numerically greater improvement in KCCQ scores in men. However, these findings were exploratory and may reflect baseline differences or sex-related variation in symptom perception rather than true differences in treatment effect (10).

Moreover, in real-world registries, there is a significant female predominance among patients undergoing percutaneous treatments for A-STR, including TriClip procedures and transcatheter valve replacement. This finding suggests a greater inclination towards minimally invasive strategies in women, possibly due to higher surgical risk and the prevalence of atrial forms, which are more amenable to percutaneous approaches (20).

### 1.4 Objectives of the review

This review summarizes current evidence on sex differences in secondary tricuspid regurgitation (TR), focusing on epidemiology, pathophysiology, guideline recommendations, procedural outcomes, and patient-reported outcomes after transcatheter tricuspid edge-to-edge repair (T-TEER). It also examines gender-related disparities in referral pathways and access to care and identifies key gaps in the available T-TEER literature.

The aim is to provide an overview of sex differences across TR phenotypes and in the clinical response to T-TEER, and to highlight gender-related disparities in referral pathways and access to care. Specifically, we aim to promote timely diagnosis and individualized therapeutic strategies to improve symptoms, quality of life, and potentially prognosis. Given that much of the available sex-stratified evidence derives from exploratory analyses and registries rather than prospectively designed sex-based trials, this review critically appraises the current literature while acknowledging its methodological limitations.

## 2 Literature search and evidence synthesis

### 2.1 Review design

This structured narrative review was developed to provide a transparent synthesis of the available evidence on sex-related differences in tricuspid regurgitation and gender-related disparities in access to transcatheter treatment. The methodological approach was informed by the SANRA recommendations for narrative reviews, while study identification and selection were reported using a PRISMA-informed flow diagram adapted to the narrative design of the review (21).

### 2.2 Database and information sources

PubMed/MEDLINE was used as the primary bibliographic database. The electronic search was supplemented by backward citation searching of eligible articles and relevant reviews. Official European Society of Cardiology/European Association for Cardio-Thoracic Surgery and American College of Cardiology/American Heart Association websites were examined to identify pertinent guidelines and consensus documents. Guidelines, consensus statements, and narrative reviews were used for clinical context and citation chaining but were not considered primary clinical studies in the PRISMA-informed selection count.

#### 2.2.1 Keywords and search strategy

The search strategy combined controlled vocabulary and free-text terms relating to three principal domains: tricuspid regurgitation; sex or gender; and transcatheter tricuspid valve intervention. Disease-related terms included “tricuspid regurgitation,” “tricuspid valve insufficiency,” “secondary tricuspid regurgitation,” “atrial secondary tricuspid regurgitation,” and “ventricular secondary tricuspid regurgitation.” Sex- and gender-related terms included “sex,” “sex differences,” “sex-specific,” “sex-related,” “gender,” “gender differences,” “gender-related,” “women,” “female,” “men,” and “male.” Intervention-related terms included “transcatheter edge-to-edge repair,” “transcatheter tricuspid edge-to-edge repair,” “T-TEER,” “TriClip,” “PASCAL,” “transcatheter tricuspid valve repair,” “transcatheter tricuspid valve intervention,” “TTVI,” “transcatheter tricuspid valve replacement,” and “TTVR.” Broader TTVI terminology was retained to avoid missing studies that reported T-TEER outcomes using an umbrella definition. The complete reproducible PubMed strategies are provided in Supplementary Table S1.

### 2.3 Research period

The literature search covered records indexed in PubMed/MEDLINE from database inception through 31 January 2026. No lower publication-date restriction was applied. The final search cutoff was selected to include the most recent relevant evidence available during manuscript revision while preserving the original scope and narrative structure of the review.

#### 2.3.1 Inclusion and exclusion criteria

Eligible publications included original clinical studies involving adults with tricuspid regurgitation that reported sex-stratified or gender-related data concerning epidemiology, clinical presentation, anatomical or pathophysiological characteristics, access to care, candidacy for transcatheter intervention, procedural outcomes, prognosis, right ventricular remodeling, residual TR, heart failure hospitalization, survival, or patient-reported outcomes. Randomized controlled trials, prospective and retrospective cohort studies, multicenter and single-center registries, and administrative database analyses were considered eligible.

Studies were excluded from the primary evidence synthesis if they involved pediatric or congenital populations, isolated case reports, preclinical or non-human investigations, conference abstracts without sufficient extractable data, or populations without relevant TR-specific or sex-stratified findings. Editorials, narrative reviews, systematic reviews, and general guideline documents were not treated as primary clinical evidence but could be used for background information and citation chaining. No language filter was applied directly to the electronic search; eligibility was determined during screening according to the availability of sufficient information for assessment.

### 2.4 Study selection and data synthesis

Records were first screened by title and abstract. Publications considered potentially relevant were subsequently assessed in detail against the prespecified eligibility criteria. For each eligible report, information was collected on study design, sample size, proportion of women, TR phenotype, intervention type, procedural success, residual TR, right ventricular parameters, mortality, heart failure hospitalization, reintervention, and patient-reported outcomes.

The searches identified 372 records: 154 through the epidemiology and sex/gender search and 218 through the transcatheter intervention and sex/gender search. After removal of 11 duplicate records, 361 unique records were screened. Of these, 312 were excluded during title and abstract assessment, and 49 were assessed in detail. Twenty-three publications were excluded after detailed assessment, leaving 26 reports for the structured narrative synthesis. The selection process is summarized in Supplementary Figure 1.

A quantitative meta-analysis was not undertaken because the available evidence included heterogeneous study designs, patient populations, TR phenotypes, intervention definitions, follow-up durations, and outcome measures. Moreover, most studies did not provide comparable adjusted sex-specific effect estimates. Statistical pooling was therefore considered potentially misleading, and the findings were synthesized narratively.

## 3 Epidemiology, clinical presentation, and access disparities

### 3.1 Epidemiological differences and clinical presentation

Population-based data indicate that tricuspid regurgitation is modestly more prevalent in women than in men. In the Framingham Heart Study, TR of at least mild severity was present in 18.4% of women and 14.8% of men. TR prevalence increased with age in both sexes. In multivariable analysis, age was associated with TR of at least mild severity (OR 1.5 per 9.9-year increase; 95% CI, 1.3–1.7), while female sex was associated with an OR of 1.2 (95% CI, 1.0–1.6) (22). More recent prospective heart failure registry data confirmed a higher prevalence of at least moderate TR in women than in men (42% vs. 29%). Severe TR was associated with mortality particularly in women with HFpEF and in men with reduced ejection fraction, suggesting that sex should be interpreted together with the underlying heart failure phenotype (23).

These data identify female sex as a potential independent risk factor for the development of the disease, likely due to a combination of anatomical, functional, pathophysiological, and hormonal factors.

The A-STR and V-STR subtypes show a different distribution between sexes, with specific prognostic implications, as detailed in the “Pathophysiology” section (*<InsFlag>*Figure 1).

**Figure 1:**
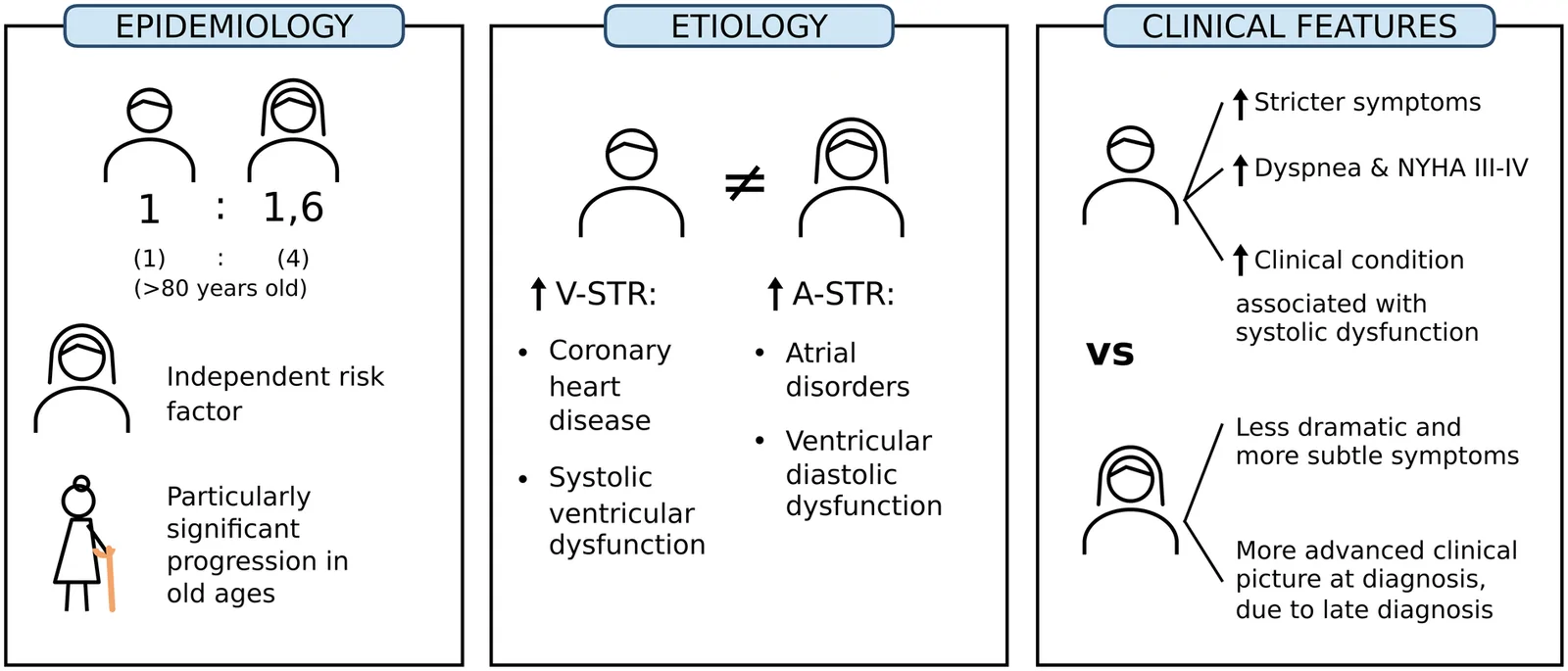
Sex-related differences in secondary tricuspid regurgitation. Epidemiology shows a higher prevalence in women, particularly in advanced age, where female sex represents an independent risk factor. Etiology differs by sex, with V-STR, mainly associated with coronary artery disease and systolic dysfunction, more frequent in men, while A-STR, linked to atrial disorders and diastolic dysfunction, is predominant in women. Clinical features also diverge: men usually present with more evident symptoms and systolic dysfunction, whereas women often show subtler manifestations, but at a more advanced stage due to delayed diagnosis.

Quantitative sex-stratified data from a multicenter cohort of 702 patients undergoing transcatheter tricuspid valve intervention are summarized in *<InsFlag>*Table 1. A-STR was more frequent in women, whereas V-STR was more frequent in men. Atrial fibrillation was highly prevalent in both sexes without a statistically significant difference. HFpEF was not reported as a separate sex-stratified diagnosis in this cohort and was therefore not inferred from preserved LVEF alone.

**Table 1:** Sex-stratified tricuspid regurgitation phenotype and selected preprocedural characteristics in a multicenter cohort undergoing transcatheter tricuspid valve intervention.

Parameter	Women (*n* = 386)	Men (*n* = 316)	*P* value
Atrial secondary TR, *n* (%)	161 (41.7)	77 (24.4)	<0.001
Ventricular secondary TR, *n* (%)	193 (50.0)	204 (64.6)	<0.001
Atrial fibrillation, *n* (%)	341 (88.3)	291 (92.1)	0.128
HFpEF diagnosis, *n* (%)	NR	NR	—
Coronary artery disease, *n* (%)	137 (35.5)	167 (52.9)	<0.001
Left ventricular ejection fraction, %	55.6 ± 10.6	51.2 ± 12.3	<0.001

Values are presented as *n* (%) or mean ± standard deviation. Percentages refer to the respective female or male subgroup. NR = not reported; A-STR = atrial secondary tricuspid regurgitation; V-STR = ventricular secondary tricuspid regurgitation; HFpEF = heart failure with preserved ejection fraction. A-STR and V-STR do not total 100% because primary and cardiac implantable electronic device-related TR were also represented. These findings describe a selected high-risk interventional population and should not be interpreted as pooled prevalence estimates.

### 3.2 Gender disparities in access to specialist assessment and transcatheter intervention

Direct TR-specific evidence on gender-related referral bias remains limited. However, in a retrospective cohort of 330 patients with severe primary mitral regurgitation meeting Class I indications for intervention, women were less likely than men to undergo multidisciplinary evaluation (57% vs. 84%) and subsequent mitral valve intervention (47% vs. 69%) (26). These findings, illustrated in *<InsFlag>*Figure 2, derive from primary mitral regurgitation rather than TR and are presented solely as indirect evidence of potential sex-related disparities in valvular referral pathways; they should not be quantitatively extrapolated to patients with TR (24).

**Figure 2:**
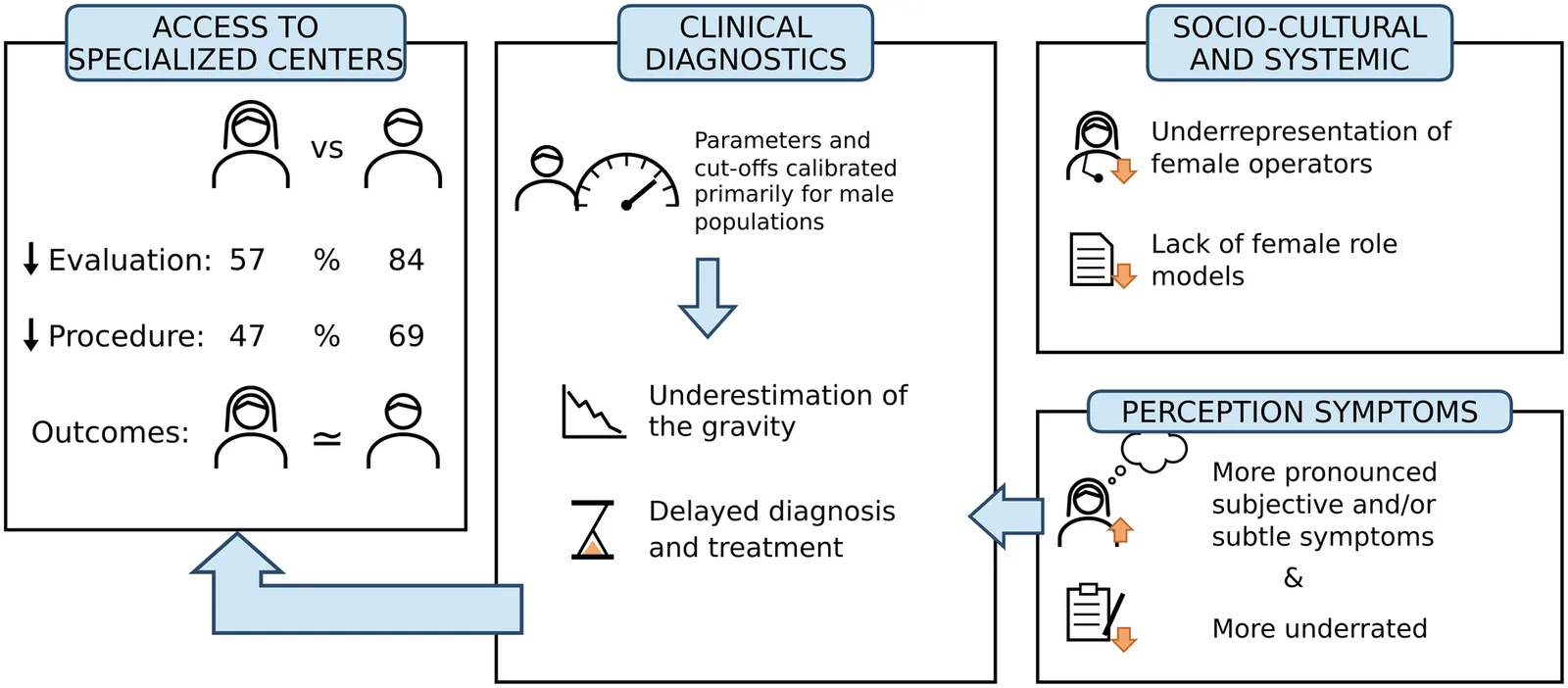
Sex- and gender-related disparities in access to specialist evaluation, clinical diagnosis, and symptom perception. The numerical values displayed in the left panel are derived from the retrospective study by Waldron et al. (24)., which included 330 patients with severe primary mitral regurgitation meeting Class I indications for intervention. In that cohort, women were less likely than men to undergo multidisciplinary evaluation (57% vs. 84%) or mitral valve intervention (47% vs. 69%). These percentages relate specifically to primary mitral regurgitation and are included solely as indirect evidence of potential sex-related disparities in valvular referral pathways; they should not be interpreted or quantitatively extrapolated as TR-specific estimates. The “Outcomes” symbol is qualitative and does not represent a specific measured endpoint or an assertion of numerical equivalence between women and men. The central and right panels are conceptual and summarize potential diagnostic, sociocultural, systemic, and symptom-perception mechanisms that may contribute to delayed diagnosis and treatment in women; no TR-specific quantitative estimates are implied.

Sex-related differences in cardiac dimensions may affect the interpretation of echocardiographic measurements when non-sex-specific reference ranges or management thresholds are applied. Women may therefore need to exhibit more advanced structural abnormalities before reaching guideline-based thresholds, potentially contributing to delayed disease recognition and specialist referral. However, validated TR-specific quantitative estimates for this potential disparity are not currently available (25).

Fortmeier et al. reported a sex-related difference in the prognostic association between TAPSE/mPAP and 2-year mortality after TTVI. However, the proposed thresholds were derived retrospectively within a single multicentre cohort using sex-stratified spline analyses and have not been externally validated. Accordingly, they should be regarded as exploratory prognostic markers and should not currently be used to determine referral timing, procedural eligibility, or treatment selection (11).

Another relevant bias concerns clinical presentation, as highlighted by the ESC/EACTS Guidelines on Valvular Heart Disease. Women often report more pronounced subjective symptoms, such as dyspnea, fatigue, or syncope. These symptoms may be underestimated, which can further delay specialist evaluation.

The limited representation of women among interventional cardiologists in structural heart disease programs in another often-overlooked issue. According to Burgess et al. (2022), only 1%–2% of operators in this field are women. Similarly low proportions have been reported in TAVR and TMVr programs. Although direct evidence linking operator gender to TR-specific outcomes is lacking, this imbalance may still influence referral pathways, patient engagement, and awareness of sex-specific disease presentation. In the context of TR, these considerations remain exploratory and warrant further investigation (26) (Figure 2).

Overall, while emerging evidence suggests the presence of gender-related disparities in access to valvular care, robust TR-specific data are still lacking, underscoring the need for dedicated studies in this field.

## 4 Pathophysiology and sex-related mechanisms

### 4.1 Anatomical and functional differences between men and women

The tricuspid valve apparatus, similarly to the mitral valve, consists of four main components: leaflets, chordae tendineae, papillary muscles, and the tricuspid annulus. The leaflets are connected to the chordae tendineae and ensure proper coaptation during the cardiac cycle, a process that is closely influenced by right ventricular (RV) performance.

Under physiological conditions, the annulus has a saddle-shaped and dynamic morphology, with its maximal dimension in mid-to-late systole. This configuration promotes optimal leaflet coaptation and a more uniform distribution of traction forces. In functional TR, loss of this geometry, together with annular flattening and progressive remodelling, impairs coaptation and increases the regurgitant orifice area.

These structural changes are clinically relevant because they may influence outcomes after TEER. Three-dimensional echocardiographic studies have shown an average annular circumference of approximately 12 ± 1 cm and an average area of 11 ± 2 cm^2^ (27).

In functional tricuspid regurgitation, the annulus loses its elliptical configuration and becomes more circular and progressively dilated. An annular dimension ≥40 mm (or ≥21 mm/m^2^) in the apical four-chamber view is considered pathological (28, 29). Three-dimensional morphological analyses have shown that the absolute annular size is on average larger in men. However, this difference disappears after indexing to body surface area (BSA), suggesting that women do not inherently have a smaller annulus, but rather one that is proportionate to body size (28).

A post-mortem anatomical study of 101 human hearts (48 male and 53 female) reported myocardial tissue in the atrioventricular annuli of all male specimens and in one female specimen; male annuli also showed greater elasticity and cellularity. These observations should not be interpreted as a universal binary distinction between male and female tricuspid annuli, because they derive from a single histological series examining atrioventricular annuli. They suggest, but do not establish, that sex-associated variation in annular composition may influence susceptibility to dilatational remodeling (13, 15).

From a functional perspective, despite similar systolic pulmonary arterial pressure (sPAP) values between sexes, women tend to maintain preserved RV systolic function more often, but with lower wall thickness (RV WT). In men, RV remodeling is generally more pronounced, with greater contractile impairment and worse prognosis (29).

A contemporary cohort of 554 patients with moderate or severe secondary TR further showed that women experienced a comparable risk of mortality or heart failure hospitalization at lower effective regurgitant orifice areas and regurgitant volumes, smaller indexed RV volumes, higher RV ejection fraction, and smaller tricuspid annular dimensions. These findings support further evaluation of sex-specific thresholds for TR severity and right-heart remodeling (30, 31).

### 4.2 Role of sex hormones, ventricular compliance, and pressure load on disease progression

Available evidence suggests that sex hormones may be associated with differences in right ventricular structure and adaptation, although these data do not derive from patients with secondary TR. In a Dutch pulmonary arterial hypertension (PAH) cohort, circulating sex-hormone profiles, including testosterone, were associated with long-term RV adaptation. In the population-based MESA-RV study, conducted in adults without clinically evident cardiovascular disease, higher estradiol levels were associated with higher RV ejection fraction and lower RV end-systolic volume among postmenopausal women using hormone therapy, whereas higher androgen levels were associated with greater RV mass and volumes (32).

Experimental PAH and pressure-overload models have also generated hypotheses involving estrogen-receptor signaling and RV remodeling, but these observations remain disease- and model-dependent (33, 34). Recent clinical data from systemic sclerosis-associated pulmonary arterial hypertension also suggest sex-related differences in right ventricular remodeling and prognosis. In a retrospective single-center cohort of 61 patients, men had a higher prevalence of right ventricular dilation and worse cardiovascular event-free and overall survival than women. However, only nine men were included, and these disease-specific findings should be regarded as exploratory and should not be extrapolated to secondary TR (35).

Direct mechanistic evidence that sex hormones influence the development or progression of secondary TR is currently absent. Importantly, PAH is characterized predominantly by pulmonary vascular disease and chronic RV pressure overload, whereas secondary TR encompasses a heterogeneous combination of atrial and annular remodeling, RV dysfunction, left-sided heart disease, pulmonary hypertension, and regurgitant volume overload. Extrapolation from PAH cohorts, population-based RV imaging studies, and animal models to secondary TR is therefore speculative. Sex-hormone signaling should consequently be regarded as a biologically plausible hypothesis rather than an established determinant of secondary TR progression, response to transcatheter intervention, or clinical outcome (36).

### 4.3 Impact of comorbidities (atrial fibrillation, heart failure with preserved ejection fraction) more prevalent in females

Tricuspid regurgitation (TR) is often linked to heart failure (HF) and atrial fibrillation (AF), which can affect its prevalence, clinical progression, and prognosis. In the general population, the estimated prevalence of moderate-to-severe TR is 3%–6%, but this can increase to 10%–29% in patients with HFpEF or HFrEF, and up to 39% in those with both AF and HFpEF (37, 38).

According to an observational multicenter analysis from the ESC-HFA registry, isolated TR was present in 5.5% of heart failure patients, whereas moderate/severe mitral regurgitation was observed in 17% and combined MR/TR in 11%. In patients with HFpEF, isolated TR was more common (OR 1.94; 95% CI 1.61–2.33), whereas isolated MR and combined MR/TR were less frequent (OR 0.42; 95% CI 0.36–0.49, and OR 0.59; 95% CI 0.50–0.70, respectively) (37).

New-onset atrial fibrillation (AF) is an important risk factor for the development of TR, particularly in women. In women, AF is more often associated with pre-existing or emerging diastolic dysfunction and with an underlying HFpEF phenotype. In this setting, TR is linked to post-capillary pulmonary hypertension, biatrial remodeling, progressive annular and RV dilation, and left ventricular diastolic dysfunction due to ventricular interdependence (39, 40).

Disease progression has been better characterized by the staging proposed by Dietz et al., which identifies four stages of secondary TR. Patients in stages 3 and 4, characterized by marked dilation and advanced RV dysfunction, had more than double the five-year mortality risk compared with those in stage 1 (HR 2.11; 95% CI 1.16–3.83). These data demonstrate that TR is not merely a marker of advanced disease, but an independent risk factor for mortality (16).

From a sex perspective, these observations are particularly relevant. Women, who are more frequently affected by HFpEF and chronic AF, often develop A-STR with an insidious progression and an unfavorable prognosis. Important management gaps remain, including underdiagnosis and undertreatment, especially in women with HFpEF. A better understanding of the interplay between HFpEF, AF, and TR may help guide future trials and support more personalized therapeutic strategies (1).

Importantly, sex-related differences in TR presentation may largely reflect differences in underlying etiology rather than intrinsic differences in procedural response. Women more frequently exhibit the A-STR phenotype, whereas men more commonly present with V-STR, often in the context of left-sided heart disease or ventricular remodeling. Therefore, sex should be interpreted together with TR phenotype rather than as an isolated determinant of treatment response. Because these phenotype and comorbidity patterns may influence procedural candidacy and post-intervention response, the next section places T-TEER within current guideline recommendations and examines the implications of treatment timing in women.

## 5 Guideline perspective and sex-specific considerations for the timing of T-TEER

### 5.1 European and north American recommendations

The 2021 ESC/EACTS Guidelines emphasized that intervention for severe tricuspid regurgitation should be considered before the development of irreversible right ventricular damage and end-organ failure and stated that transcatheter treatment may be considered in symptomatic patients with severe secondary TR who are considered inoperable, are anatomically eligible, and are evaluated by a Heart Team at an experienced Heart Valve Centre when symptomatic or prognostic improvement can reasonably be expected (41).

The 2024 Clinical Consensus Statement of the Heart Failure Association and the European Association of Percutaneous Cardiovascular Interventions of the ESC should be regarded as a clinical consensus update rather than a formal focused guideline update; it reinforces early identification, phenotype-based assessment, optimization of volume status, multimodality evaluation of right ventricular function, invasive haemodynamic assessment when appropriate, and referral to a Heart Failure Clinic and Heart Valve Centre before severe right ventricular dysfunction or irreversible renal and hepatic injury develops, while acknowledging that the optimal timing of intervention remains uncertain (42).

By comparison, the 2020 ACC/AHA Guidelines do not provide a dedicated recommendation for tricuspid transcatheter edge-to-edge repair, reflecting the limited transcatheter evidence available when the document was developed; they state that surgery is reasonable in symptomatic patients with severe functional TR, particularly when associated with atrial fibrillation and atrial annular dilation, provided that severe right ventricular dysfunction, pulmonary hypertension, and advanced hepatic or renal damage are absent (Class IIa) (41).

More recently, the 2025 ESC/EACTS Guidelines upgraded the role of transcatheter tricuspid valve intervention, stating that it should be considered to improve quality of life and right ventricular remodelling in high-risk patients with severe symptomatic TR despite optimal medical therapy, in the absence of severe right ventricular dysfunction or pre-capillary pulmonary hypertension (Class IIa, Level of Evidence A); this recommendation applies to transcatheter treatment broadly, including T-TEER when the valve anatomy is suitable (43).

Taken together, these documents show a progressive evolution from cautious consideration of transcatheter treatment in inoperable patients toward a more evidence-based role for TTVI in symptomatic high-risk patients; nevertheless, patient selection should still integrate symptoms, TR severity and mechanism, anatomical suitability, right ventricular function, pulmonary haemodynamics, end-organ status, expected quality-of-life benefit, and individual patient preferences (41, 43).

### 5.2 Timing in women and the potential need for sex-specific criteria

None of the current European or North American recommendations provides sex-specific thresholds for referral or intervention in TR, and although the 2025 ESC/EACTS Guidelines include a dedicated discussion of sex-related considerations in valvular heart disease, they do not establish TR-specific criteria for women and men (41, 43).

This absence may be clinically relevant because women are diagnosed with significant TR at an older age and more frequently present with isolated or left-sided-valvular-disease-related TR, whereas men more commonly exhibit TR associated with left ventricular dysfunction, suggesting that identical referral triggers may not fully capture sex-related differences in disease phenotype and progression (16).

In women, earlier referral should not be interpreted as intervention based on sex alone; rather, it should involve structured surveillance and timely Heart Valve Centre assessment when severe atrial secondary TR persists despite appropriate management of atrial fibrillation, HFpEF, and volume status, particularly in the presence of progressive right atrial or right ventricular remodelling, impaired RV–pulmonary artery coupling, increasing diuretic requirements, worsening functional capacity, or early renal and hepatic congestion (16, 42).

Although exploratory observational data suggest that sex may modify the prognostic association between TAPSE/mPAP and mortality after TTVI, the proposed cut-offs were internally derived from a single retrospective multicentre cohort and have not been externally validated. They should therefore not be interpreted as validated referral, eligibility, or treatment-selection criteria for T-TEER (11). Future prospective studies should therefore evaluate whether sex-specific or sex-indexed criteria—including indexed tricuspid annular and right ventricular dimensions, RV strain and RV–pulmonary artery coupling, symptom and patient-reported outcome burden, and early markers of systemic congestion—improve the timing and selection of women and men for T-TEER; until such criteria are prospectively validated, sex should inform clinical assessment but should not independently determine procedural candidacy (11, 20). The following section examines whether these considerations translate into sex-related differences in procedural and clinical outcomes after T-TEER.

## 6 Procedural response and clinical outcomes

This section focuses exclusively on objective clinical and echocardiographic outcomes following TEER, including procedural success, survival, heart failure hospitalization, residual TR, and RV function.

### 6.1 Success rates of the triClip procedure in women vs. men

In a multicenter German–Swiss cohort study by Fortmeier et al. (2023), 702 patients underwent TTVI for severe TR, including 386 women and 316 men. Procedural success rates were comparable between sexes, with a reduction of at least one TR grade in 90.9% of women and 91.8% of men (*p* = 0.796). As these findings derive from observational data, they should be interpreted cautiously and considered hypothesis-generating (11).

Although procedural success rates were comparable between women and men, anatomical suitability should be assessed independently of sex. Absolute tricuspid annular dimensions are generally smaller in women, but these differences are substantially attenuated after indexing to body surface area, and no sex-specific device-sizing criteria have been validated. In a retrospective cohort, estimated eligibility for TTVI was similar in women and men (10.8% vs. 9.2%), although approximately three quarters of both groups were considered ineligible because of advanced disease; these estimates did not represent actual device-specific screening failure rates. Moreover, although the A-STR phenotype that is more frequent in women may be associated with less leaflet tethering, advanced annular dilatation can produce a large coaptation gap and reduce T-TEER feasibility. Therefore, TR phenotype should be considered together with coaptation-gap width, leaflet anatomy, and imaging quality when selecting the most appropriate device strategy (17, 28, 44).

### 6.2 Sex-based comparison of objective outcomes

Registry-based analyses suggest that TEER outcomes are broadly similar in women and men. In the analysis by Alachkar et al., women had a numerically higher in-hospital procedural success rate than men (95% vs. 88%), although the difference was not statistically significant (*p* = 0.177). In-hospital mortality also appeared comparable between sexes (45).

More recent real-world analyses reinforce these observations. In a United States administrative analysis of 1,960 T-TEER procedures, women represented 61.7% of patients and, after adjustment, had lower inpatient mortality (adjusted OR 0.43; 95% CI 0.22–0.82) and fewer major complications (adjusted OR 0.69; 95% CI 0.49–0.98) than men (46).

Consistently, the TRI-SPA registry included 310 patients undergoing T-TEER, 70% of whom were women. Procedural success was comparable in women and men (90.7% vs. 89.3%; *p* = 0.71), with no significant sex-related difference in the 12-month composite of all-cause mortality, heart failure hospitalization, or tricuspid valve reintervention. Sustained TR reduction and functional improvement were also comparable between sexes (47).

In the sex-specific analysis of the TriValve registry by Scotti et al., 556 patients underwent TTVI, including 316 women (56.8%) and 240 men. At 1 year, freedom from all-cause mortality was similar in women and men (80.9% vs. 77.9%; *P* = 0.56), as were heart failure hospitalization (*P* = 0.36), NYHA functional class III or IV (*P* = 0.17), and residual TR >2 + at the latest follow-up (*P* = 0.42) (20).

Overall, registry-based and subgroup analyses suggest a broadly comparable objective clinical response in women and men. However, because most analyses were not pre-specified for sex and may be underpowered, these findings should be interpreted as exploratory.

In the Fortmeier multicenter cohort, 2-year survival was likewise comparable between women and men (69.9% vs. 63.7%; *P* = 0.144) (11).

In the IPTW-adjusted analysis by Scotti et al., the 556 patients undergoing TTVI were compared with 2,072 patients receiving medical therapy alone. TTVI was associated with improved 1-year survival compared with medical therapy in both women (adjusted HR 0.45; 95% CI, 0.23–0.83; *P* = 0.01) and men (adjusted HR 0.42; 95% CI, 0.18–0.89; *P* = 0.03) (20).

A single-center study of 105 patients (63 women, 42 men) confirmed similar one-year mortality rates between sexes (25.9% vs. 24.3%; *p* = 0.863), as well as HF hospitalization rates (37.5% vs. 40.5%; *p* = 0.768) (45).

The role of biomarkers, particularly NT-proBNP, remains underexplored in the post-TTVI setting due to limited sex-stratified data. Studies in HF patients (not specific to TTVI) have shown that women tend to present with higher NT-proBNP levels than men at similar clinical severity (48). This difference is likely influenced by biological factors such as sex hormones, body composition, and fat distribution, which modulate both production and metabolism of natriuretic peptides (49). However, in the specific context of TTVI, robust sex-specific data are still lacking (*<InsFlag>*Table 2).

**Table 2:** Selected characteristics, study-specific procedural or early efficacy endpoints, and clinical outcomes from major studies and registries of tricuspid transcatheter edge-to-edge repair. Procedural metrics are reported according to the definitions used in the respective studies and should not be interpreted as a common or directly comparable endpoint.

Study/registry	Population	Women	Study-specific procedural or early efficacy endpoint*	Selected clinical outcomes
TRILUMINATE Pivotal RCT – primary randomized cohort	350	54.9%	Implant success: 98.8% (170/172 attempted procedures); TR ≤ moderate at 30 days: 87.0% (140/161 patients with evaluable echocardiography) (50)	No apparent between-group difference in death or tricuspid valve surgery or in heart failure hospitalization at 1 year
Tri.Fr	300	63.7%	Implant success: 97.3% (144/148 patients undergoing T-TEER) (9)	No significant between-group difference in major cardiovascular events or cardiovascular mortality at 1 year
bRIGHT registry	511	56.0%	Acute procedural success: 92% in the prespecified primary analysis cohort of the first 200 patients; implant success: 99% (504/511); full-cohort procedural success: 91% (451/496 patients with evaluable TR severity) (51)	Single-arm estimates: all-cause mortality 15.1% and heart failure hospitalization 15.3% at 1 year; NYHA class I–II increased from 21% to 75%
TRI-SPA registry	310	70.0%	Study-defined procedural success: 90.7% in women versus 89.3% in men (*P* = 0.71) (47)	12-month composite endpoint: 16.3% in women versus 27.5% in men; adjusted HR 0.61 (95% CI, 0.31–1.18; *P* = 0.142)
Fortmeier et al.	702	55.0%	TR reduction by ≥1 grade: 90.9% in women versus 91.8% in men (*P* = 0.796); residual TR ≤ II/V: 77.5% versus 77.2% (11)	Two-year survival: 69.9% in women versus 63.7% in men (*P* = 0.144)
TriValve registry	556	56.8%	Not available (7)	One-year freedom from all-cause mortality: 80.9% in women vs. 77.9% in men; HF hospitalization, NYHA class III–IV, and residual TR >2 + were also comparable between sexes

*Procedural and early efficacy outcomes are reported according to the terminology, denominator, and assessment time point used in each original study. In TRILUMINATE, implant success denotes successful device implantation among attempted procedures, whereas TR ≤ moderate at 30 days is a separate echocardiographic efficacy endpoint. In Tri.Fr, implant success denotes successful device implantation among patients undergoing T-TEER. In bRIGHT, acute procedural success was defined as successful device implantation with a core laboratory-assessed TR reduction of at least one grade at discharge; the full-cohort procedural-success analysis used the 30-day TR grade when the discharge assessment was unavailable. In TRI-SPA, procedural success was based on residual TR ≤2 + at discharge. In the Fortmeier study, TR reduction by at least one grade and residual TR ≤ II/V were reported separately. These outcomes differ in definition, denominator, adjudication, and assessment time point and should not be directly compared across studies.

The variability of NT-proBNP related to sex, age, and BMI highlights the importance of individualized assessment of valvular hemodynamic burden, especially in women. This is critical to prevent over- or underestimation of right-sided HF and to improve the selection of patients for TTVI.

It has also been suggested that women may commonly exhibit a phenotype of preserved RV reserve but reduced tolerance to afterload, potentially impacting post-procedural clinical outcomes (29).

### 6.3 Analysis of high-risk female subgroups and results in national registries

In the retrospective multicentre cohort reported by Fortmeier et al., sex-stratified spline analyses identified TAPSE/mPAP values of <0.612 mm/mmHg in women and <0.434 mm/mmHg in men as thresholds associated with increased 2-year all-cause mortality after TTVI. These cut-offs were derived internally within the same cohort and were not tested in an independent population. They should therefore be regarded as exploratory, hypothesis-generating prognostic estimates rather than validated biological boundaries or criteria for referral, procedural candidacy, or patient selection. Prospective external validation is required before their application in clinical practice (11, 52). The interpretation of TAPSE-based indices should also account for measurement reproducibility. In a standardized study of 100 patients with systemic sclerosis undergoing echocardiographic screening for pulmonary hypertension, TDI S’ showed substantially greater inter-operator reproducibility than TAPSE. However, the cohort had preserved right ventricular function and did not include patients with TR or undergoing TTVI; these findings therefore support a multiparametric assessment of right ventricular function rather than the use of TDI S’ as a substitute for validated TR-specific thresholds (53).

RV–PA coupling should also be interpreted dynamically after intervention, because abrupt TR reduction can increase effective RV afterload and occasionally cause transient RV dysfunction or clinically relevant afterload mismatch. The incidence and possible sex-specific susceptibility to this phenomenon remain unknown (54).

Objective endpoints capture procedural and hemodynamic response, whereas patient-reported outcomes reflect symptom burden and perceived benefit. Considering both is essential for a comprehensive assessment of TEER response.

## 7 Quality of life and patient-centered outcomes (subjective response to post-triClip improvement: assessment using the Kansas city cardiomyopathy questionnaire (KCCQ) and other tools)

This section addresses patient-reported and symptom-based outcomes, including quality-of-life measures and subjective functional improvement after TEER.

Randomized 1-year trials demonstrated substantial reductions in TR severity and consistent improvements in symptoms and quality of life after T-TEER, but did not show significant reductions in mortality or heart failure hospitalization. In both TRILUMINATE and Tri.Fr, the benefits of the hierarchical composite endpoints were driven predominantly by patient-reported outcomes. In the prespecified 2-year TRILUMINATE follow-up, T-TEER reduced recurrent heart failure hospitalizations compared with medical therapy, whereas all-cause mortality remained similar (9, 10, 50).

The KCCQ has traditionally been used to evaluate patient-reported disease status in heart failure, but it has also proven useful for assessing subjective symptom perception in patients with TR (55).

In a cohort study including 2,693 patients from both single-arm and randomized trials, the KCCQ showed good psychometric properties in patients with severe TR undergoing TTVI. Score changes were small in clinically stable patients, whereas lower scores were associated with higher mortality and a greater risk of heart failure hospitalization. The instrument was therefore confirmed as a valid and reliable tool in this setting, including in the female subgroup, which represented 61.6% of the study population (56, 57).

In the TRILUMINATE pivotal primary randomized cohort (*n* = 350), the mean KCCQ change from baseline to 1 year was +12.3 points in the T-TEER group and +0.6 points in the medical-therapy group, corresponding to a between-group difference of +11.7 points (95% CI, 6.8–16.6; *P* < 0.001). A subsequent analysis of the full randomized cohort (*n* = 572) found a ≥ 15-point KCCQ improvement in 52.3% of patients receiving T-TEER and 23.5% of controls (*P* < 0.0001). Women represented 54.9% of the primary randomized cohort and 58.9% of the subsequent full randomized cohort. Exploratory sex-specific analyses suggested that women treated with T-TEER were more likely to achieve a clinically meaningful KCCQ improvement than women receiving medical therapy. However, the trial was not designed or powered to evaluate sex–treatment interactions, and these findings should therefore be interpreted as hypothesis-generating.In the Tri.Fr randomized trial, which included 300 symptomatic patients with severe TR, T-TEER with TriClip was associated with improved patient-reported outcomes compared with optimized medical therapy alone. At 1 year, the mean within-group KCCQ change from baseline was +15.9 points in the T-TEER plus optimized medical therapy group and +0.4 points in the optimized medical therapy group. The reported between-group difference was +14.5 points (*P* < 0.001). In addition, 74.6% of treated patients reported improvement in patient global assessment, compared with 39.5% in the medical-therapy group (*P* < 0.001). Women represented 64% of the study population. In subgroup analyses, women also showed significant improvement after TriClip, with a mean KCCQ increase of +15.2 points versus +0.3 in controls and better patient global assessment (73.8% vs. 40.2%) (9).

In the bRIGHT prospective multicenter single-arm registry, which included 511 patients, the paired within-group mean KCCQ change from baseline to 1 year was +19 ± 26 points among the 335 patients with available paired assessments (*P* < 0.0001). Overall, 56.2% of patients achieved a clinically meaningful improvement of at least 15 points. Because bRIGHT did not include a control group, this result represents a within-group change and should not be interpreted as a treatment effect relative to medical therapy. The proportion of patients in NYHA class I–II also increased from 21% at baseline to 75% at follow-up. Women represented approximately 56% of the study population. Subgroup analyses showed similar symptomatic and quality-of-life improvement in women, consistent with the overall findings (51) (*<InsFlag>*Table 3).

**Table 3:** One-year changes in Kansas city cardiomyopathy questionnaire overall summary score after tricuspid transcatheter edge-to-edge repair. In the randomized TRILUMINATE and Tri.Fr trials, within-group changes are presented separately for the intervention and control groups, together with the reported between-group difference. The bRIGHT value represents a paired within-group change in a single-arm registry and therefore has no between-group comparator. Owing to differences in study design, statistical analysis, denominator, and handling of missing data, these estimates should not be directly compared across studies.

Study	Design and comparator	Mean KCCQ change from baseline to 1 year in the T-TEER group	Mean KCCQ change in the control group	Reported between-group difference
TRILUMINATE – primary randomized cohort (50)	Randomized; T-TEER versus medical therapy	+12.3 points	+0.6 points	+11.7 points (95% CI, 6.8–16.6; *P* < 0.001)
Tri.Fr (9)	Randomized; T-TEER plus optimized medical therapy versus optimized medical therapy alone	+15.9 points (SD 30.1)	+0.4 points (SD 25.7)	+14.5 points (*P* < 0.001)
bRIGHT (51)	Prospective single-arm registry	+19 ± 26 points; paired analysis, *n* = 335	Not applicable	Not applicable

Some observations suggest that women may report greater subjective functional limitation despite comparable objective parameters. This apparent discrepancy may reflect differences in symptom perception, comorbidity burden (e.g., HFpEF or atrial fibrillation), or health-status reporting patterns rather than differences in procedural efficacy.

Although subgroup analyses from TRILUMINATE and Tri.Fr suggest consistent quality-of-life improvement in women, these analyses were not prospectively designed or powered to detect sex–treatment interaction effects and should therefore be interpreted as exploratory. Overall, available randomized and observational data indicate that TEER is associated with significant symptomatic and quality-of-life benefit in both women and men.

In contrast, objective clinical outcomes—including mortality, residual TR, and heart failure hospitalization—appear broadly comparable between sexes across studies. The potential divergence between objective endpoints and patient-reported outcomes may reflect differences in symptom perception, comorbidity burden, or disease phenotype rather than true variation in treatment efficacy. Given that most sex-specific findings derive from subgroup and registry-based analyses, these observations should be considered hypothesis-generating and require prospective validation.

Although available data suggest meaningful symptomatic benefit in both sexes, the evidence base remains limited by important methodological constraints.

## 8 Gaps in knowledge and future directions

Despite advances in TR treatment, significant knowledge gaps remain concerning the role of sex in disease onset, progression, and therapeutic response. In the Framingham Heart Study, the prevalence of TR of at least mild severity was modestly higher in women than in men (18.4% vs. 14.8%), and female sex was associated with an adjusted OR of 1.2 (95% CI, 1.0–1.6) (22). Sex-specific evidence in the interventional setting remains largely derived from observational registries, including the TriValve registry and the Fortmeier cohort, and from exploratory subgroup analyses of randomized trials. Furthermore, women more frequently present with isolated TR or TR associated with left-sided valvular disease, which may remain underdiagnosed and lead to delayed intervention (16, 58).

The data sources described are inherently limited by potential selection bias, residual confounding, lower female representation in some cohorts, and absence of prospective sex-stratified randomization. Consequently, observed similarities or differences in procedural success, survival, and quality-of-life improvement between women and men should be interpreted as hypothesis-generating rather than definitive evidence of sex-neutral treatment effects (45).

A quantitative meta-analysis was not considered methodologically appropriate because the available studies differed substantially in design, patient selection, TR phenotype, device or intervention definition, follow-up duration, and outcome reporting. In addition, most sex-specific findings were derived from exploratory or *post hoc* subgroup analyses, and comparable adjusted effect estimates were not consistently available across studies. Pooling these heterogeneous estimates could therefore produce a summary effect that would be difficult to interpret clinically.

Assessment of sex differences is further complicated by historically low female representation in many studies and a lack of endpoints designed to capture sex-specific symptomatic and functional features. Valvular anatomical differences, higher prevalence of comorbidities (HFpEF, chronic AF), and differential right ventricular response to pressure overload necessitate study designs that are sensitive to these variables.

Future trials should incorporate pre-specified sex-stratified analyses and patient-centered endpoints (e.g., KCCQ, quality of life, functional capacity) to evaluate the efficacy and safety of percutaneous therapies in both women and men. The systematic integration of a gender perspective in clinical research will help clarify the impact of sex on procedural and clinical outcomes and guide more personalized and equitable strategies for TR management. Moreover, whether sex-related anatomical and pathophysiological differences differentially influence outcomes across distinct TTVI platforms (edge-to-edge repair versus valve replacement) remains largely unexplored and warrants dedicated prospective investigation. Alternative echocardiographic measures of right ventricular–pulmonary arterial coupling also warrant evaluation in TR. In a multicenter registry of 347 patients with incident pulmonary arterial hypertension, a lower FAC/PASP ratio was independently associated with higher 1-year and 5-year all-cause mortality. However, FAC/PASP has not been validated in TR or TTVI populations, and the available analysis was not designed to establish sex-specific thresholds (59).

## 9 Clinical and organizational implications

Beyond these research priorities, the possibility of sex- and gender-related inequalities in access to care has immediate clinical and organizational implications. Patients with severe TR should be referred for specialist assessment before the development of advanced right ventricular dysfunction or irreversible renal and hepatic congestion, particularly when progressive right atrial or right ventricular remodelling, worsening functional capacity, recurrent heart failure hospitalization, or increasing diuretic requirements are observed. In women, greater awareness of the frequent association between atrial secondary TR, atrial fibrillation, and HFpEF may help reduce the risk of symptom under-recognition or misattribution. Nevertheless, referral decisions should remain based on objective clinical, imaging, and disease-stage criteria rather than on sex alone (42, 43).

Dedicated referral pathways should connect primary care, community cardiology, heart failure and atrial fibrillation clinics, echocardiography laboratories, and local hospitals with experienced Heart Valve Centres. Standardized referral criteria and predefined clinical and imaging red flags may facilitate timely and reproducible access to specialist assessment. At the Heart Valve Centre, a multidisciplinary Heart Team should confirm TR severity and mechanism, optimize medical therapy, evaluate right ventricular function and pulmonary haemodynamics, assess anatomical suitability, and determine the appropriate timing of T-TEER or alternative treatments. Structured post-assessment feedback and follow-up plans should also be provided to the referring centre (42, 43). Beyond referral and procedural decision-making, structured follow-up should consider access to exercise-based cardiac rehabilitation where clinically appropriate. Evidence from broader cardiovascular populations indicates that women may encounter lower referral, participation, and completion rates because of caregiving responsibilities, psychological factors, socioeconomic constraints, and limited access to services. Technology-enabled, home-based, multidisciplinary, and family-centered models may help address these barriers; however, dedicated evidence on rehabilitation adherence after T-TEER is currently lacking (60).

Prospective TR and TTVI registries should be specifically designed to identify potential disparities across the entire care pathway. Rather than establishing entirely separate registries for women and men, existing and future registries should ensure adequate representation and prespecified sex-stratified reporting of referral, treatment selection, procedural outcomes, patient-reported outcomes, heart failure hospitalization, and mortality. Where feasible, gender-related determinants—including socioeconomic barriers, caregiving responsibilities, symptom-reporting patterns, and access to specialist care—should be collected separately from biological sex. The Valvular Heart Disease in Women Registry provides an emerging organizational model by evaluating guideline adherence, time from symptom onset to tertiary-centre presentation, time from diagnosis to guideline-directed treatment, and procedural complications according to sex and gender (61, 62).

These registries could also support the development of candidate sex- and gender-oriented key performance indicators. Potential indicators include the proportions of women and men with severe TR referred to a Heart Valve Centre; the intervals from diagnosis to referral, Heart Team assessment, and intervention; the proportion receiving guideline-concordant multimodality imaging; representation in TTVI registries and clinical trials; completion of patient-reported outcome measures; and procedural and clinical outcomes stratified by sex. These indicators should be adjusted for age, TR mechanism and severity, comorbidity burden, and anatomical eligibility. Because validated TR-specific gender-oriented KPIs are not currently available, these measures should initially be regarded as research and local quality-improvement tools rather than mandatory sex-based treatment targets (61, 62).

## 10 Conclusion

TR in women remains frequently underdiagnosed and undertreated, despite growing evidence that transcatheter edge-to-edge repair with TriClip is associated with significant symptomatic improvement. Available data suggest broadly comparable procedural success and quality-of-life benefit in women and men, although most sex-specific findings derive from observational studies or exploratory subgroup analyses.

Gender-related disparities still affect access to specialist evaluation, timing of referral, and trial representation, and may contribute to delays in care.

In clinical practice, a more personalized approach may improve management. This approach should integrate TR phenotype (A-STR vs. V-STR), established measures of right ventricular function, common comorbidities such as AF and HFpEF, sex-aware interpretation of biomarkers, and structured follow-up including patient-reported outcomes. The incremental clinical value of sex-specific RV–PA coupling thresholds remains investigational and should not guide referral or procedural candidacy until externally validated.

Future studies should clarify whether the sex-related patterns observed after T-TEER are also present with other transcatheter tricuspid platforms, including emerging valve replacement technologies. Early referral may be especially relevant in women with A-STR, before progression to advanced right ventricular dysfunction.
