# Supplementary material for: Sex differences and gender-related disparities in transcatheter tricuspid edge-to-edge repair for secondary tricuspid regurgitation: toward a personalized approach in cardiac care
Source: Front Cardiovasc Med. 2026 Oct 30;13:1831980. doi: 10.3389/fcvm.2026.1831980 (PMC13760338; doi:10.3389/fcvm.2026.1831980)
Supplement: Supplementary file 1 [file Supplementaryfile1.docx]

 **Supplementary Figure S1.** PRISMA-informed flow diagram of study identification and selection. The diagram was adapted for a structured narrative review and represents the selection of primary clinical evidence. Guidelines, consensus documents, and background reviews were considered separately for contextual discussion and citation chaining. TR, tricuspid regurgitation; T-TEER, transcatheter tricuspid edge-to-edge repair.
